# What Explains Successful or Unsuccessful Postural Adaptations to Repeated Surface Perturbations among Older Adults?

**DOI:** 10.3390/ijerph182212069

**Published:** 2021-11-17

**Authors:** Jimmy Falk, Viktor Strandkvist, Irene Vikman, Mascha Pauelsen, Ulrik Röijezon

**Affiliations:** Department of Health, Education and Technology, Luleå University of Technology, 97187 Luleå, Sweden; viktor.strandkvist@ltu.se (V.S.); Irene.Vikman@ltu.se (I.V.); mascha.pauelsen@ltu.se (M.P.); ulrik.roijezon@ltu.se (U.R.)

**Keywords:** balance, postural control, surface perturbation, older people, falls

## Abstract

As we age there are natural physiological deteriorations that decrease the accuracy and flexibility of the postural control system, which increases the risk of falling. Studies have found that there are individual differences in the ability to learn to manage repeated postural threats. The aim of this study was to investigate which factors explain why some individuals are less proficient at adapting to recurrent postural perturbations. Thirty-five community dwelling older adults performed substantial sensory and motor testing and answered surveys regarding fall-related concerns and cognitive function. They were also subjected to three identical surface perturbations where both kinematics and electromyography was captured. Those that were able to adapt to the third perturbation were assigned to the group “Non-fallers” whereas those that fell during all perturbations were assigned to the group “Fallers”. The group designation dichotomized the sample in a hierarchical orthogonal projection of latent structures— the discriminant analysis model. We found that those who fell were older, had poorer physical performance, poorer strength and longer reaction times. The Fallers’ postural control strategies were more reliant on the stiffening strategy along with a more extended posture and they were less skillful at making appropriate feedforward adaptations prior to the third perturbation.

## 1. Introduction

The risk of falling increases with age and frailty [1]. Aside from potential serious physical injuries [2], related issues such as fall-related concerns might lead to withdrawal from social [3] and physical activities [4], with negative effects on physical fitness [3] and quality of life [5]. To avoid falls and maintain ambulatory independence, we rely on well-functioning postural control. Accurate perception of the environmental- and task-specific constraints relies on adequate central processing of sensory information from mainly visual, vestibular and somatosensory systems. The perceived demands and experience of similar situations enables planning and preparation of postural actions called feedforward control. While performing an action, the constant sensory feed enables continuous corrections and reactions of our movements to manage the task in the current environment, called feedback control [6].

Different circumstances require different strategies to avoid falls. During new and unpredictable tasks, a common strategy is to increase stiffness and thereby increase impedance (i.e., resistance to movement) at the joints. As skill improves and tasks become more predictable, well-coordinated feedback and feedforward control strategies develop [7]. Postural control strategies to keep balance are often defined as ankle strategy and hip strategy, where the base of support is stationary (i.e., in place strategies), or the stepping or reaching strategy (i.e., change in support strategies) [6]. The ankle strategy is suitable for smaller postural challenges. If the ankle is unable to produce a matching reaction to a destabilizing threat (e.g., when standing on a compliant or moving surface), the hip strategy is more appropriate [8]. However, these definitions are not straightforward, as combinations of these strategies are often used [9]. If the postural integrity is further challenged, either the stepping or reaching strategy might be warranted to avoid falling [6].

As we age there are changes in our postural control that might have a negative effect on ambulatory independence. The age-related degenerations of the sensory systems result in proprioceptive loss [3], impaired vision [10], and loss of vestibular function [11]. Additionally, the ability to change the momentary reliance on sensory systems (i.e., sensory re-weighting) deteriorates [12]. Motor performance is affected by age-dependent cerebral atrophy [13], and loss of muscle mass and function [14]. These natural age-related changes decrease the accuracy and flexibility of the postural control system [15], and make postural control less automated and cognitively more taxing [13,16]. Psychological factors, such as fall-related concerns and cognitive capacity, have shown to be interlinked [17] and correlated with worse postural control [18,19,20] and increased risk of falling [21,22]. In addition to these internal changes, polypharmacy is a fall risk factor with potentially negative drug-drug or drug-disease interactions [23].

Common reasons for falls among the older population are slips and trips. A common way to investigate the strategies used for a slip or trip are various experimental surface perturbations. Older adults show poorer postural control strategies when subjected to a novel surface perturbation compared to young adults [24,25], and are more than twice as likely to fall [26]. However, when the task is repeated several times, older adults adapt to more appropriate postural reactions [24,25,27,28] with similar learning rates as younger adults. Successful adaptations result from both feedforward [28,29] and feedback control adjustments [28,30]. However, there are individual differences in the skill of adapting to repeated postural challenges [25]. Some individuals are very skillful and never fall, some learn to make appropriate adaptations after a few trials, while some seem unable to make these adaptations and continue to fall trial after trial. To the knowledge of the authors, no previous study has made a comprehensive investigation of why some individuals struggle to adapt to a predictable postural challenge.

The aim of this study was to investigate which psychological, sensory, motor and postural control variables explain which individuals are unable to adapt to a proficient postural control strategy after repeated surface perturbations.

## 2. Materials and Methods

### 2.1. Participants

Forty-five adults over the age of 70 that fulfilled the inclusion criteria of: being able to read 100 pt. large block letters, stand unassisted for 30 s, and understand simple instructions were recruited to the study. Informed consent was obtained from all participants involved in the study. The study was conducted according to the guidelines of the Declaration of Helsinki and was approved by the Institutional Regional Ethical Review Board in Umeå, Sweden (ref no. 2015-182-31, 2 June 2015).

### 2.2. Test Protocol

#### 2.2.1. Setting

This project is part of the Balancing Human and RoboT (BAHRT) project, which is an inter-disciplinary project at the Luleå University of Technology. The test protocol was conducted at the “Human Health and Performance Lab–movement science” at the Luleå university of technology, Luleå, Sweden.

#### 2.2.2. Motor Control Strategies

The participants’ ability to manage and adapt to postural challenges was tested by three repeated surface perturbations. The participants stood on a six-degrees of freedom platform (CKAS Mechatronics Pty Ltd., Tullamarine, Australia), wearing a safety harness around the chest and handheld straps which gave no support unless the participant was about to fall. They were informed that the surface would move, but not when or how. The platform was programmed to translate anteriorly for 8 cm at a velocity of 10 cm/s, then tilt 6° at a velocity of 11°/s and then tilt back to level surface. The test was repeated three times with a few seconds of rest in between. The participants were encouraged to keep an in-place strategy during the perturbations. If they stepped or needed support from the safety harness/straps that trial was considered a fall. According to the outcome, the sample was divided in two groups: one group who failed to keep an in-place strategy for every perturbation (Fallers), and one group who were able to successfully manage at a minimum the third perturbation (Non-fallers). To decide which strategies the participants used in anticipation and as a reaction to the perturbation, the data recordings were divided over two periods. The feedforward period measuring −100–0 ms before platform perturbation onset and the feedback period recording from the onset of platform movement until the platform stopped at its most anterior and tilted position. To discover the different strategical adaptations between those who were able to adapt to the perturbations from those that were not, the data were extracted from the first and last perturbation. During the surface perturbation test, kinematics and electromyography (EMG) were synced and recorded in the software Qualisys track manager (Qualisys Inc., Gothenburg, Sweden).

##### Kinematics

**Figure A1:**
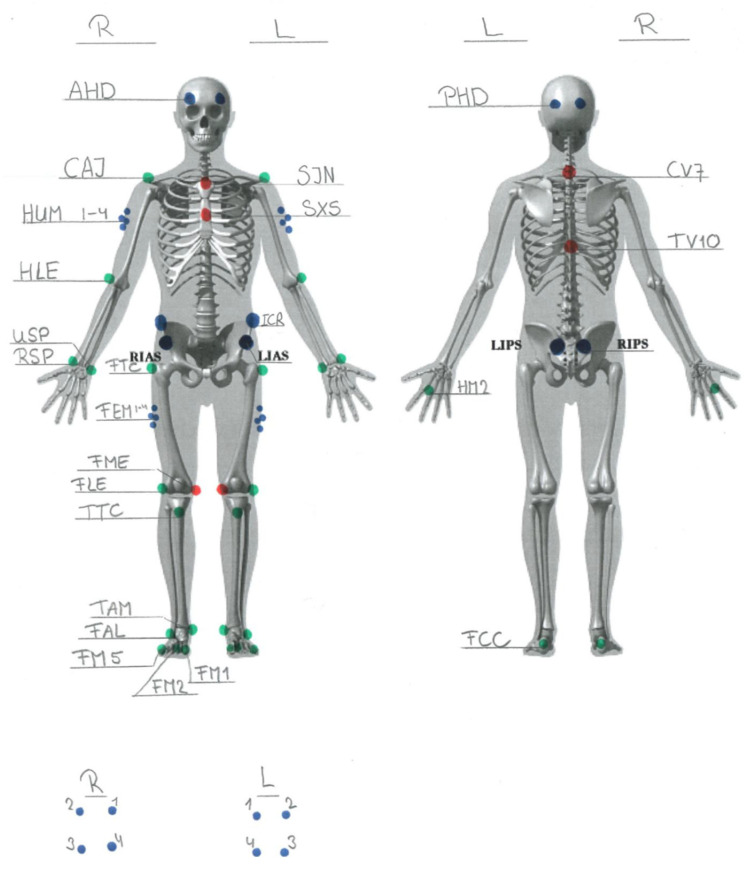
Illustration of the placement of the reflective markers for the full-body model in the Qualisys track manager software, this figure is reprinted with permission [48].

The kinematics from the surface perturbation test were recorded by the Qualisys Pro Reflex capture system (Qualisys Inc., Gothenburg, Sweden) with a sample frequency of 200 Hz. A full body model was built by placing 60 reflective markers on specific body landmarks with one marker positioned at each corner of the platform, see Appendix A, Figure A1. The kinematic data quantified joint angles for the hips, knees and ankles, where the angle for the left and right side was averaged for the respective joint. The angle between the pelvis and the 7th cervical vertebra quantified sagittal spinal motion. Prior to the perturbation test, the joint angles were captured during a quiet stance trial in erected standing posture. This position was used to normalize the joint angles for each participant and the angle of each joint at this position was considered zero degrees, respectively. For the feedforward period, the average joint angles were calculated for 100 ms, where a positive value indicated flexion of the back, hip, and knee, as well as plantar flexion of the ankle, whereas a negative value indicated the opposite movement of that joint. For the perturbation period, the maximum flexion and extension angles for each joint were calculated.

##### Electromyography

EMG of the tibialis anterior and medial gastrocnemius was sampled bilaterally by Ag–AgCl dual surface EMG electrodes with a fixed 2 cm inter-electrode spacing (Noraxon Inc., Scottsdale, AZ, USA). The electrodes were placed- and the skin was prepared according to the recommendations of SENIAM [31]. EMG was recorded by the Noraxon DTS 16 channel wireless EMG system (Noraxon Inc., Scottsdale, AZ, USA) with a sample frequency of 3000 Hz. The muscle onset of tibialis anterior after the start of the perturbation was visually detected in the QTM software. The EMG feedback period was defined as the period between the tibialis anterior onset and the halt of the platform. The EMG-data for both tibialis anterior and gastrocnemius were bandpass filtered 20–500 Hz, the root-mean squared with a 50 ms sliding window and normalized to a maximum isometric contraction. The EMG of the left and right-side muscles were averaged into one signal for tibialis anterior and one signal for gastrocnemius. The processed EMG of the two opposing muscles were used to compute a co-contraction index (CCI) [32], where the average value over the feedforward and feedback period was calculated, respectively.

The kinematic and EMG data from the surface perturbation test were extracted with a Matlab script, a link to which script is found at https://github.com/LTU-Human-Health-and-Performance/BAHRT (accessed on 14 November 2021).

#### 2.2.3. Medication

The number of medicines taken daily by the participants was documented.

#### 2.2.4. Psychological Instruments

The psychological domain was investigated with six instruments investigating distinct aspects of fall-related concerns, and one cognitive screening tool. The participants answered the Fear of falling scale, where they were asked if they were afraid of falling, answering on a Likert scale ranging from 1 = No, to 4 = Yes, very [33]. A four-question battery, based on a study by Yardley et al., evaluated the participants concerns about the consequences of a potential fall [34]. The participants answered the following questions on a Likert scale from 1 = not at all worried, to 4 = yes, very worried: “If you were to fall, are you worried to injure yourself?”, “If you were to fall, are you worried to stay helpless on the floor?”, “If you were to fall, are you worried to need more help afterwards?” and “If you were to fall, are you worried to be a burden afterwards?” They also answered the Falls Efficacy Scale-International (FES-I), which is a valid and reliable 16 item survey where each question is answered on a four-point Likert scale. The total score ranges between 16 and 64, where a score > 23 indicates high concerns of falling [35]. The cognitive function was screened with the Mini Mental Test (MMT). The results are summarized on a scale from 1 to 30, where a higher number indicates higher cognitive function. The most frequent cut-off score to indicate cognitive impairment is a score of ≤23 [36].

#### 2.2.5. Sensory Testing

Adjusted bi-ocular visual acuity was tested with an NFD chart, with the individual standing 5 m from the chart. The score was reported according to the decimal system, where 1.0 is considered normal vision and a score < 1.0 indicates worse vision. The vestibular system was screened by having participants wear Frenzel glasses to assess the presence of nystagmus during active and passive rotations of the neck, as well as when looking up, down, left, and right. Joint position sense was tested for the knee and ankle in the Biodex system 3 (Biodex Medical Systems, Inc., Shirley, NY, USA). For the knee, the participant was seated, relaxed, and with 90° hip flexion and 90° knee flexion with the lower leg hanging down. Then the participant, blindfolded, would actively extend the knee to 30° where the Biodex locked the joint angle for five seconds, and the participant was asked to remember that position. Then after reverting to the starting position, the participant would try to actively reposition the joint to 30°. The mean absolute error of three trials was calculated. The same procedure was done for the ankle joint by trying to reposition the ankle from 20° plantar flexion to 5° dorsiflexion.

#### 2.2.6. Strength Testing

Muscle strength was tested with the Biodex system 3, where the participants applied maximal isometric force against a pad at the end of a static lever. Hip extension was performed prone with 90° knee flexion with the pad distally at femur. Hip abduction was tested in side lying with a straight knee and the pad resting against the distal femur. Knee flexion and extension was tested in a seated position, with the knee joint at 30° of flexion with the pad positioned just proximal of the malleolus. Ankle joint torque was tested in a reclined position, with the seat at 55°, a limb support under the distal femur and the lower legs parallel to the floor, creating a slight angle at the knees and a neutral ankle angle. The feet were strapped to a pedal to test both plantar- and dorsiflexion torque. Each test was performed three times under strong encouragement from the test leader. The highest torque for each participant and muscle group was normalized by dividing torque with the body height of the participant.

#### 2.2.7. Functional Testing

Reaction time was tested with a personal computer and custom-made software. The test involved a black screen which suddenly turned green after a random time elapse of between 5 and 10 s. An audible signal occurred simultaneously with the color change and the task was to react to these stimuli as fast as possible by hitting the space key on the keyboard. The mean time in milliseconds (ms) over five trials was calculated. The participants performed the short physical performance battery (SPPB), which is a well-established instrument that assesses standing balance, gait, and chair stands. The score ranges from 0 (worst performance) to 12 (best performance). It is an instrument with good reliability and validity to assess physical performance among older individuals [37].

#### 2.2.8. Postural Sway Testing

The standing postural sway of the participants was tested in four trials of quiet stance during different conditions. The participant stood on a Kistler force plate (Kistler, Winterthur, Switzerland) either with or without a six cm thick compliant balance-pad (AIREX, Sins, Switzerland), with open or closed eyes. The stance was standardized so that the distance between the first metatarsals was equal to 75% of the distance between the anterior superior iliac spines, with a self-chosen rotational angle of the feet. Each trial was recorded for 30 s. The Kistler software allows for a sampling rate of 1500 or 3000 Hz. In order to be able to sync the signals with those of the kinematics (sampled at 200 Hz), a sampling rate of 3000 Hz was used. The postural sway data was filtered with a lowpass Butterworth filter with a cutoff at 10 Hz. Then, for each of the four trials, the area of the smallest ellipse that fitted 95% of the data point swarm was calculated by using a principal component analysis (PCA).

### 2.3. Statistics

Statistical testing was performed with SPSS statistics 28 (IBM corp., Armonk, NY, USA). The subject characteristics group difference between Fallers and Non-fallers are presented in median and interquartile range and then were tested with the Mann-Whitney U-test (Table 1). The inter-trial difference of the kinematics and electromyography for each group were tested with the Wilcoxon signed-rank test. The data were imported to SIMCA 15 (Sartorius AG, Göttingen, Germany). The grouping variable Faller/Non-faller was set as “Class-ID”. Variables measuring similar constructs were inserted in PCA hierarchal base models. The scores of these models were included in the hierarchal top model along with the remaining independent variables. The top model, in order to discover which variables could explain the group belonging of the participants, was an Orthogonal Projection of Latent Structures—Discriminant Analysis (OPLS-DA) model. A variable was considered to have significant weight according to the model if the confidence interval of the coefficient did not include zero. To test the generalizability of the final top model, a permutation plot was generated.

### 2.4. Ethical Considerations

Written informed consent was obtained from all participants involved in the study. The study was conducted according to the guidelines of the Declaration of Helsinki and approved by the Institutional Regional Ethical Review Board in Umeå, Sweden (ref no. 2015-182-31, 2 June 2015).

## 3. Results

### 3.1. Participants

Out of the 45 recruited participants, five were considered as too unfit to perform the perturbation test. Additionally, five individuals were excluded from analysis due to technical errors that lead to substantial data loss. The vestibular test identified only one participant with positive signs for nystagmus during the provocation tests, hence they were excluded from the analysis. The final sample consisted of 14 men and 21 women, of which 30 individuals successfully kept an in-place strategy during the third perturbation and were thereby grouped as Non-fallers, and five individuals did not, and were grouped as Fallers. All those who failed the in-place task during the third perturbation had also failed in the previous perturbations. Descriptive data are presented in Table 1.

### 3.2. Main Results

Four separate PCA models were generated to represent different constructs and were set as hierarchal base models:

- The base model “Fall-related Concerns”, containing the variables “Are you afraid of falling?”, and the four items of consequence concern, and FES-I, produced one principal component with an explained variance (R2Y) of 57.7% and a predictive value (Q2) of 31.2%.
- The four trials of quiet stance balance concluded the “Balance” base model, made of one principal component with a R2Y of 57.1% and Q2 of 19.3%
- “Strength” is the base model enclosing all strength tests for the lower extremity, which resulted in a one component model with an R2Y of 69.5% and Q2 of 63.1%.
- The joint position sense tests for both left and right knee and ankle produced the base model “Joint Position Sense” with one principal component with an R2Y value of 56% and a Q2 value of 14.7%.

**Figure 1:**
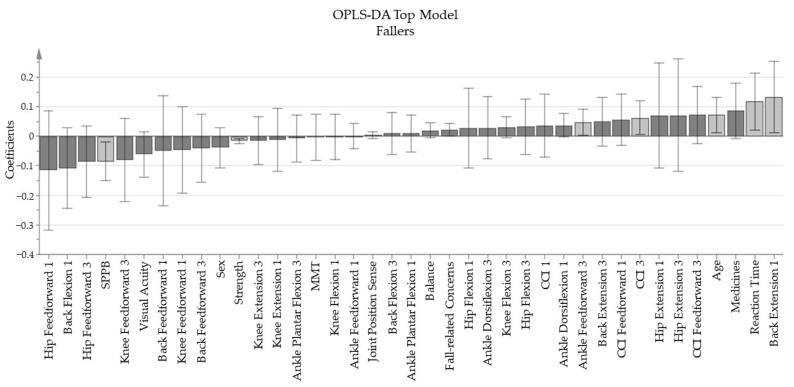
The coefficients of the final top model for the group “Fallers”. Bars to the left of the plot with a negative direction shows that fallers have lower values for those variables compared to the non-falling group; the opposite relations occur for the bars to the right with a positive direction. Variables with a significant weight to the model are indicated with a light grey bar.

**Figure A2:**
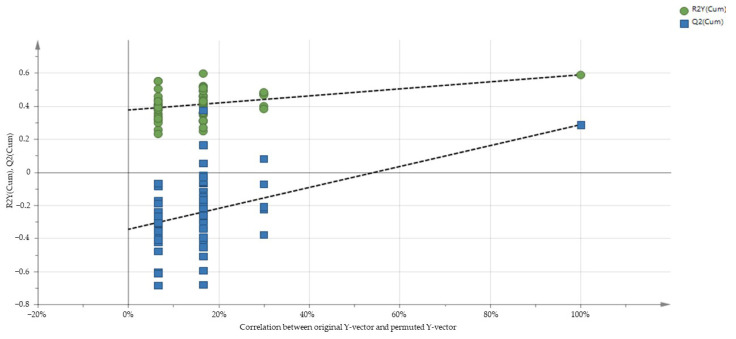
Permutation plot of the final OPLS-DA top model. This analysis recreates 50 models with permuted Y-values, shown to the left. The original model´s R^2^Y and Q^2^ values are shown to the right from which a regression line is drawn through the permuted R^2^Y and Q^2^ values to the vertical axis. A strong model has higher Q^2^ and R^2^Y values than all of the permuted models and/or the Q2 regression line to intercept the vertical axis below zero. The plot shows the intercept of the regression line of the R2Y at 0.38 and Q2 at −0.35.

The OPLS-DA top model showed a clear separation of the two groups, with an explained variance (R2Y) of 59% and a predictive value (Q2) of 28.8%. The coefficients of the model are shown in Figure 1. The permutation plot showed that the final top model is weak (Appendix A, Figure A2).

The coefficients show that the group that is not able to adapt to a proficient postural strategy (i.e., the Fallers), have poorer leg muscle strength, are older, have a longer reaction time and show lower scores on the SPPB. During the feedforward period Fallers stood with more ankle plantar flexion prior to the third perturbation. As a feedback response, Fallers extended the back more during the first perturbation and stiffened the ankle joints more during the third perturbation.

**Figure 2:**
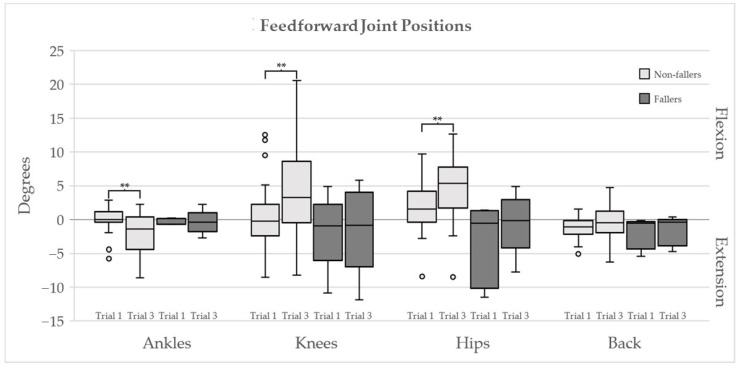
Boxplots of the feedforward joint positions prior the first and the third perturbation trials for the non-fallers (light grey) and fallers (dark grey). The positive values indicate knee, hip, and back flexion as well as ankle plantar flexion. Difference between the two trials for the respective groups were tested with the Wilcoxon signed rank test; double asterisks indicate significant difference *p* < 0.001.

**Figure 3:**
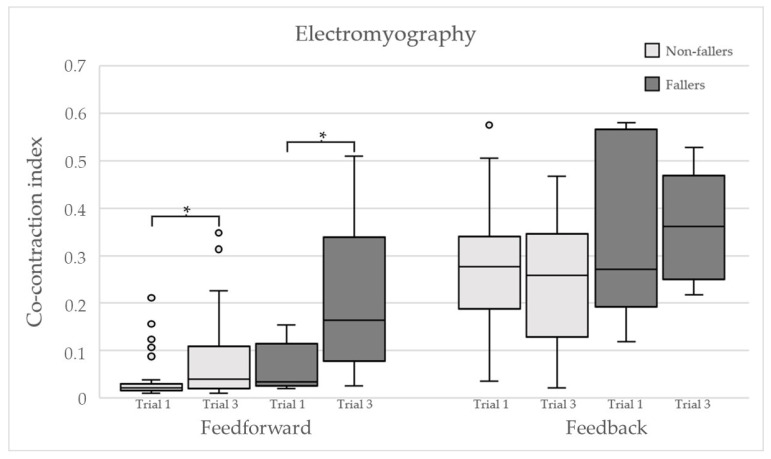
Co-contraction index for both groups during the feedforward and the feedback period for both trials. An asterisk indicates significant inter-trial difference for the group, tested with the Wilcoxon signed-rank test.

The feedforward kinematics are presented in Figure 2. The median and interquartile range of the feedback kinematics are presented in Table 2. The electromyography data are shown in Figure 3.

## 4. Discussion

The aim of the study was to investigate which factors explain why some individuals are successful and others unsuccessful at adapting an adequate postural control strategy to repetitive surface perturbations. We found that 14% of the participants in this study were not able to make proficient adjustments to manage recurring surface perturbations. This group, according to the final OPLS-DA top model, were older and had lower muscle strength, slower reaction time and lower physical performance levels based on the SPPB. For a novel perturbation they responded with more back extension. For a recurrent perturbation they stood with less dorsiflexion prior to the perturbation and responded with higher levels of agonist-antagonist co-contractions during the task.

The findings show that both physiological capacity and postural control strategies affect the capacity of older individuals to adapt to repeated surface perturbations. The results align with the results of a recent systematic review and meta-analysis by Jehu et al. that investigated the risks of recurrent falls (two or more falls per year) by dividing risk factors in seven domains: balance and mobility, environmental, psychological, medical, medication, sensory and neuromuscular, and sociodemographic. Four domains could successfully predict recurrent falls. Sensory and neuromuscular testing proved to be one of the two most important domains for predicting recurrent falls. We quantified the sensory and neuromuscular systems with strength testing of the lower extremities, the computer-based reaction time test, an assessment of visual acuity and joint position sense of the knees and feet. The reaction time test proved to be the neuromuscular variable that had the most impact on the final top model. This is interesting as it is quite different from the perturbation test. The reaction time test is reliant on the reaction and processing of a visual and auditory stimuli to produce a motor command to press a button using the upper extremity. In contrast, during the perturbation tests the reaction time is dependent on the proprioceptors in the lower extremities to register a movement, sending afferent signals to the spinal cord that via reflex arcs sends efferent signals to the muscles to contract, without any conscious central processing of the signal. One could speculate that the reaction time test might be an indicator of general neurological vigor. As none of the strictly sensory tests showed significant importance for the model, but lower extremity strength did, both groups seem to have equal capacity regarding input from sensory systems, but the fallers have poorer ability to execute an effective motor response due to inadequate reaction and muscle function.

Along with sensory and neuromuscular testing, Jehu et al., found that medication was the most influential factor with regard to recurrent falls. We examined the number of medications, which did not prove to be significant for the final top model. But independent significance testing showed a significant difference between the groups, where the non-faller group had a median of two and the falling group had a median of six medications. This difference dichotomizes the groups according to the common guidelines that more than four medications are associated with increased incidence of falls, recurrent falls, and fall-related injuries [23]. The number of medicines probably reflect both the negative consequences polypharmacy has on safe mobility e.g., drug-drug interactions, and that more frail individuals are more dependent on medications [23].

Jehu et al. found psychological factors to be the third most influential domain with regard to recurrent falls. Neither the Fall-related concerns base model nor the MMT showed a significant weight to the top model. The testing of the individual variables of the Fall-related concerns base model also did not show a significant difference between the groups. This result is contrary to our expectation, as fall-related concerns have repeatedly shown correlations with altered postural control [18,19,20]. The MMT was also used to measure the cognitive capacity of the participants, which was non-significant for the top model and the individual significance testing. This sample had high scores and low variance, possibly due to the inclusion criteria, underlining critiques that MMT is a rather crude test with a ceiling effect [36]. Interestingly, the reaction time test could be argued to also measure cognitive fitness, as central processing speed is correlated with cognitive function [38]. Moreover, for repeated reaction time testing, increased intra-individual reaction time variability (IIV) have shown strong associations with poorer cognitive function. Both IIV and reaction time mean have shown a predictive value of cognitive function five years ahead [39]. By extension, due to the relationship between cognitive function and falls, a positive relationship of IIV and falls has been found. Thus, it has been suggested that IIV (and maybe reaction time mean) testing may detect deteriorations linked to gait impairment earlier than standard gait assessment tools [40]. Reaction time tests are easily administrated and time efficient and could be a valuable instrument for fall risk assessments.

Jehu et al., found that balance and mobility was the fourth most influential domain. Our results also acknowledge the importance of this domain as those that were unskillful at adapting to the recurrent surface perturbations had lower SPPB scores. This was rather expected as the SPPB is an instrument specifically developed to assess the physical capacity amongst older individuals. Although the base model balance did not prove to be significant for the top model, when inspecting Table 1, the 95% ellipse for Fallers was significantly larger for Fallers only in the trial with unstable surface and open eyes. Of the four quiet stance balance trials, the trial with unstable surface with eyes open is the most similar to the platform perturbation test. Hence, it is reasonable and in line with the principle of specificity that fallers also have larger sway in similar situations.

Further exploratory analysis of the pattern of the final top model (Figure 1), including the non-significant independent variables, shows that fallers stand more erect prior the perturbation, and respond to the perturbation with a general extension strategy in the back and hips and have higher levels of co-contractions in the lower legs, both prior and in response to the first and the last perturbation. However, this pattern analysis is not based purely on robust data, but it might inspire future research questions. The patterns make sense as a more extended and rigid body will be less stable during perturbations [41].

The Non-fallers were more skillful at predicting the pending demands for repeating perturbation, using appropriate feedforward strategies. When inspecting Figure 2, the Non-fallers significantly increased their flexion strategy in the hips, knees and ankles in the third compared to the first perturbation. Consequently, their reactive joint motions in the third perturbation are reduced, as seen in Table 2. This is an exquisite example of the reciprocal behavior of how adequate feedforward control lowers the demands on feedback control [42].

Previous research found that the stiffening strategy diminishes as surface perturbations are repeated [30]. This was not seen in the three trials of our experiment. To the contrary, Figure 3 showed increased feedforward stiffening, especially among fallers. Moreover, the top model showed high correlations with stiffening and falls for trial three. This could have several explanations. The stiffening strategy is associated with a fear of falling [18,43,44,45,46]. Therefore, it makes sense that those who are unable to make appropriate postural adjustments feel a lack of control and possible fear during the task they´ve failed several times. An alternative explanation related to motor learning is that stiffening is used as a learning strategy to limit the degrees of freedom and free cognitive resources to the most pressing variables for the motor task [6]. According to this hypothesis, increasing the stiffening strategy for a task that you´ve failed might be an attempt to gain control in a task that is currently too overwhelming to handle.

We must recognize that in this study we considered a step or gripping the safety straps due to the perturbation as a fall. This is common in laboratory studies but not ecologically accurate, as a stepping strategy can be a successful strategy to avoid falls, which are used more commonly among those at higher risk of falling [47]. Consequently, in studies where some participants’ go-to strategy is regarded as a fail, they might have more problems with using a different strategy effectively.

The final top model came out with a good explanation of the variance in the data and predictability. However, when testing the robustness of the model, the permutation plot showed that the model is weak (Figure A2). This implies that the model is unlikely to predict the dependent variable very well for new observations, and that the results should be interpreted with caution. The sample consisted of 30 individuals that were able to manage the third perturbation and only five individuals that were not. A larger sample with more fallers would probably strengthen the model. This is not an easy task, as the safety of the participants is of the highest importance. Five participants either declined to perform the perturbation test, or were assessed by the test leader as not able to perform the test safely. These excluded individuals would have been valuable for the study and may have added to the number in the Fallers group.

Further studies that compare the sensory and motor systems as well as the applied strategies for different postural tasks are warranted. We also hope to see how different reaction time tests can be used in fall risk assessments and interventions.

## 5. Conclusions

The findings of this experimental study show that higher age, poorer physical performance, strength and reaction time are internal qualities that explain the reduced ability to adapt adequately to repeated surface perturbations among people of older age. With aspect of postural strategy during the task, those who fell used more of a stiffening strategy along with a more extended posture and did not make the required feedforward adaptations of flexing the hips, knees and ankles prior to a known surface perturbation. This demonstrates that both internal variables of physical functions and postural control strategies affect the capacity of older individuals to adapt a proficient response to repeated surface perturbations.

## Figures and Tables

**Table 1:** Descriptive data of both groups are presented in median and interquartile range.

	Non-Fallers	Fallers	Sig.
**Descriptive data**			
Sex (Male/Female)	13/17	1/4	0.627 ^a^
Age (years)	74 (71–76)	77 (75–78)	0.170 ^b^
**Height (cm)**	**167 (161–177)**	**155 (153–160)**	** 0.014 ** ^b^
Weight (kg)	71.5 (64–83)	75 (70–77)	0.873 ^b^
**Number of medicines**	**2 (1–4)**	**6 (4–7)**	**0.018** ^b^
** Psychological instruments **			
Afraid of falling	1 (1–2)	1 (1–2)	0.766 ^b^
Concerns of injury	1 (1–2.75)	2 (2–4)	0.141 ^b^
Concerns of staying helpless on the floor	1 (1–1)	1 (1–2)	0.506 ^b^
Concerns of needing more Help	1 (1–1)	1 (1–3)	0.421 ^b^
Concerns of becoming a burden	1 (1–1)	2 (2–3)	0.054 ^b^
FES-I	19 (17–23)	23 (20–24)	0.299 ^b^
MMT	29 (27–30)	28 (28–29)	0.477 ^b^
**Sensory testing**			
Visual acuity	0.8 (0.7–0.9)	0.7 (0.7–0.7)	0.054 ^b^
JPS Knee (degrees)	4.65 (3.85–6.79)	3.2 (3–6)	0.450 ^b^
JPS Foot (degrees)	3.85 (2.43–5.07)	6 (4–7.2)	0.062 ^b^
** Strength testing **			
Hip Extension torque (Nm)	49.8 (37.0–66.0)	37.3 (21.0–43.7)	0.090 ^b^
Hip Abduction torque (Nm)	51.8 (34.8–71.4)	40.8 (36.2–41.7)	0.509 ^b^
**Knee Extension torque (Nm)**	**84.5 (67.3–111.7)**	**61 (58.6–65.5)**	**0.038** ^b^
Knee Flexion torque (Nm)	61.9 (53.8–84.7)	50.1 (38.6–53.3)	0.099 ^b^
Ankle Dorsiflexion torque (Nm)	21.7 (18.3–25.5)	19.6 (15.7–2.0)	0.203 ^b^
**Ankle Plantar Flexion torque (Nm)**	**88.0 (63.2–107.5)**	**57.7 (40.4–60.4)**	**0.010** ^b^
** Functional testing **			
**Reaction Time (ms)**	**365 (327–414)**	**442 (439–448)**	** 0.012 ** ^b^
SPPB	11.5 (11–12)	10 (9–11)	0.054 ^b^
** Postural sway testing **			
Stable Eyes Open (cm^2^)	0.93 (0.71–1.73)	2.95 (1.24–3.57)	0.086 ^b^
Stable Eyes Closed (cm^2^)	1.25 (0.86–1.95)	2.81 (1.31–3.43)	0.232 ^b^
**Unstable Eyes Open (cm^2^)**	**4.95 (4.15–6.37)**	**11.08 (8.83–12.44)**	** 0.001 ** ^b^
Unstable Eyes Closed (cm^2^)	12.2 (9.12–19.32)	19.11 (18.48–21.14)	0.137 ^b^

The strength tests are presented in their raw value, Newton-metre (Nm). ^a^ Fishers exact test. ^b^ Mann Whitney U-test. Bold text indicates significant group difference. Abbreviations: FES-I = Falls Efficacy Scale—International; MMT = Mini Mental Test; JPS = Joint Position Sense; SPPB = Short Physical Performance Battery.

**Table 2:** Range of motion for each joint during the feedback period for the first and third trial are presented in median and interquartile range for the respective group.

Maximum Joint Angle (Degrees)	Non-Fallers n = 30			Fallers n = 5		
	Trial 1	Trial 3	Sig.	Trial 1	Trial 3	Sig.
**Ankle Plantar Flexion**	**0.80 (0.61–2.32)**	**2.63 (1.69–4.10)**	** 0.002 **	**0.79 (0.73–1.17)**	**1.85 (1.34–3.40)**	** 0.043 **
Ankle Dorsiflexion	6.55 (5.00–8.39)	6.48 (5.06–7.37)	0.480	7.47 (6.91–7.58)	6.70 (5.69–9.60)	0.893
**Knee Flexion**	**12.50 (11.66–16.68)**	**8.77 (5.48–11.92)**	** 0.001 **	14.01 (13.89–14.69)	8.15 (7.86–10.30)	0.225
Knee Extension	0.23 (0.16–0.31)	0.15 (0.11–0.26)	0.495	0.15 (0.14–0.30)	0.17 (0.00–0.34)	0.686
**Hip Flexion**	**7.67 (5.30–11.88)**	**3.23 (1.64–5.53)**	** <0.001 **	12.26 (8.54–19.32)	5.03 (2.43–6.29)	0.080
Hip Extension	0.04 (0.01–0.28)	0.19 (0.06–1.19)	0.139	0.35 (0.11–0.83)	2.14 (0.45–4.19)	0.686
**Back Flexion**	**2.61 (2.08–3.64)**	**1.51 (0.88–2.04)**	** <0.001 **	1.90 (0.43–1.91)	1.64 (1.00–3.13)	0.225
Back Extension	0.13 (0.01–0.61)	0.11 (0.03–0.52)	0.754	2.04 (1.69–2.56)	0.70 (0.17–2.49)	0.138

The Wilcoxon signed rank test was used to establish if the groups significantly changed their postural control strategy between the trails; significant inter-trial difference are marked in bold text.

## Data Availability

Data can be available upon request to the authors.
